# Severe Cervicothoracic Kyphoscoliosis in Neurofibromatosis - A Failure Of Posterior-Only Instrumentation: A Case Report

**DOI:** 10.5704/MOJ.2003.015

**Published:** 2020-03

**Authors:** EZF Soh, MH Muhamad-Ariffin, A Baharudin

**Affiliations:** Department of Orthopaedics and Traumatology, Universiti Kebangsaan Malaysia, Cheras, Malaysia

**Keywords:** kyphoscoliosis, neurofibromatosis, anterior and posterior stabilisation

## Abstract

Treatment of severe spinal deformities associated with neurofibromatosis has proven to be challenging. An 11-year-old girl, with neurofibromatosis and severe cervicothoracic kyphoscoliosis, was initially treated with posterior instrumentation and fusion. Implant failure developed within a year, requiring an anterior stabilisation and fusion with a non-vascularised fibular strut graft for better stability and increased likelihood of achieving union. The posterior instrumentation was removed due to its prominence and wound breakdown. Following the removal of the posterior implant, the fibular graft fractured. The patient was maintained on a cervical collar until union was achieved. Posterior spinal fusion alone in severe spinal deformities in neurofibromatosis has a high risk of failure. A combined anterior and posterior fusion may increase the chance of success, with better stability and union rate.

## Introduction

Spinal deformities are often reported in neurofibromatosis patients and can be of dystrophic or non-dystrophic type^1^. Correcting the dystrophic curve has proven to be challenging due to the high rate of failure and pseudarthrosis^2, 3^. Literature has recommended using both anterior and posterior fusion in a severe dystrophic curve to increase the chance of success^1, 2, 4^. Utilising fibula strut graft as anterior support in the correction of spinal deformities has been well reported. However, there is a risk of graft fracture. A newer technique of using vascularised fibula graft may have a higher rate of an union, but it was found to be technically more demanding^5^.

## Case Report

**Fig. 1:**
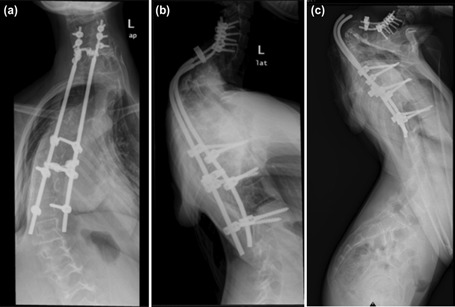
(a, b) Radiographs of the spine showing posterior spinal instrumentation and fusion in situ. (c) Broken rods at the cervicothoracic junction one year later.

An 11-year-old girl, with neurofibromatosis, first noticed a spinal deformity at the age of 7 and was diagnosed with severe cervicothoracic kyphoscoliosis. She defaulted follow-up and presented to us four years later with worsening of the spinal deformity associated with neurological deficit. She had bilateral weakness of the lower limbs with diminished sensation from the level of C5 and below. Computerised tomographic and magnetic resonance imaging showed that she had severe cervicothoracic kyphoscoliosis and ductal ectasia from C7 to T3 level. Posterior instrumentation and fusion in situ from C5 to T12 levels with decompression was performed (Fig. 1a, 1b) A year after the operation, she complained of difficulty of lifting the chin and noticed cracking sound at the thoracic region. A radiograph showed that there was an implant failure, with broken rods at the cervicothoracic junction. (Fig. 1c)

**Fig. 2:**
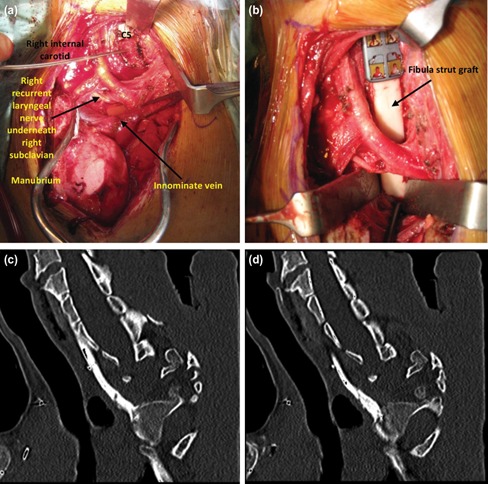
(a) Intra-operative picture showing anterior cervicothoracic stabilisation via manubrio-sternotomy. (b) Harvested fibula strut graft being placed over anteriorly at C7 to T4 level. (c, d) Computerised tomography images showing the fractured fibula graft after the removal of posterior instrumentation.

We proceeded with anterior cervicothoracic stabilisation, approaching via bilateral manubrio-sternotomy (Fig. 2a). A fibula strut graft was harvested and fixed over C7 to T4 and stabilised with an anterior cervical plate (Fig. 2b) The broken rods were also exchanged.

**Fig. 3:**
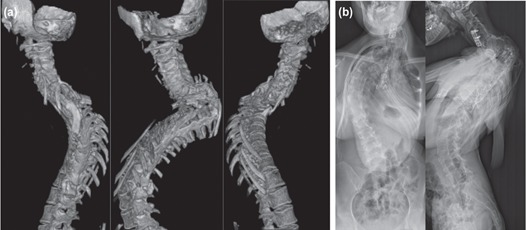
(a) Computerised tomography images showing fusion of the instrumented level. (b) Radiographs of the whole spine showing no further progression of spinal deformity.

Two years later, she developed wound breakdown over the posterior thoracic region due to the prominence of the implant. The posterior instrumentation was subsequently removed to promote wound healing. However, two months after the removal of the posterior instrumentation, the fibula graft fractured as the spinal fusion had not been completed fully (Fig. 2c, 2d). She was put on a hard cervical collar until fusion occurred completely (Fig. 3a).

She was followed up for more than six years since complete fusion occurred and was last seen six months ago with no new complaint. Recent radiographs showed no further progression of deformity (Fig. 3b). She had good recovery of her neurological deficit, with the power of bilateral lower limb at an MRC grade 4 to 5 and with intact sensation. She was able to ambulate independently.

## Discussion

In neurofibromatosis, the spinal deformities could be classified into dystrophic or non-dystrophic. Dystrophic changes included rib “pencilling”, spindling of the transverse process, scalloping of vertebral bodies, foraminal enlargement and paravertebral soft tissue tumour. Dystrophic scoliosis in neurofibromatosis often was a short-segment angular curve over the thoracic region with primary involvement of five or fewer vertebrae. The more severe dystrophic changes in the vertebral bodies were associated with a more rapid deterioration of curve, whereas the non-dystrophic spinal deformities behaved like those without neurofibromatosis^1^.

Most literature advocated for both anterior and posterior fusion in severe dystrophic scoliosis^1, 2, 3^. Rate of pseudoarthrosis in posterior spinal fusion alone in dystrophic scoliosis was reported high from 38-60%^3, 4^ Crawford recommended anterior disc excision and bone graft followed by posterior arthrodesis with instrumentation if the scoliotic curve was more than 80° or when there was a kyphosis of more than 50°^2^. Other authors recommended both anterior and posterior spinal fusion when kyphosis was more than 95°, or the apical vertebra was at a level below T83.

The failure rate of posterior fusion alone in dystrophic kyphotic curves of 50° or more was 64% -72%^4^. There was an average progression of the curve of 12.7° in posterior fusion, and more than one procedure was required to achieve solid posterior fusion^4^. Even with combined anterior and posterior fusion in dystrophic scoliosis, there was still a failure rate of 7.5%^2^.

Anterior strut graft had been used as anterior support in the treatment of spinal kyphosis, using either the fibula or the rib. However, in non-vascularised bone graft, osteocytes in the strut graft died immediately when implanted. The resorption process weakened the graft resulting in a higher risk of fracture. Vascularised fibula graft was reported with better mechanical properties with preservation of viable osteocytes, resulting in an earlier bone union, better biomechanical strength and stiffness. In Streitz’s series of 16 patients using vascularised fibular strut graft for anterior spinal fusion in spinal kyphosis, all cases achieved union in three to eight months with no reported graft fracture^5^.

In our patient, the initial posterior only instrumentation and fusion failed. We subsequently performed anterior cervicothoracic stabilisation with fibula strut graft but were still unable to achieve spinal fusion as the posterior instrumentation had to be removed early due to wound breakdown.

We are highlighting this case for the difficulty in managing severe dystrophic scoliosis and stressing the importance of both anterior and posterior stabilisation for better fusion.

## References

[1] Calvert PT, Edgar MA, Webb PJ (1989). Scoliosis in neurofibromatosis. The natural history with and without operation.. J Bone Joint Surg Br..

[2] Crawford AH (1989). Pitfalls of spinal deformities associated with neurofibromatosis in children.. Clin Orthop Relat Res..

[3] Shen JX, Qiu GX, Wang YP, Zhao Y, Ye QB, Wu ZK (2005). Surgical treatment of scoliosis caused by neurofibromatosis type 1.. Chin Med Sci J..

[4] Sirois JL (1990). 3rd, Drennan JC. Dystrophic spinal deformity in neurofibromatosis.. J Pediatr Orthop..

[5] Streitz W, Brown JC, Bonnett CA (1977). Anterior fibular strut grafting in the treatment of kyphosis.. Clin Orthop Relat Res..

